# Immune Checkpoint Blockade in Gastrointestinal Cancers: The Current Status and Emerging Paradigms

**DOI:** 10.4103/JIPO.JIPO_1_20

**Published:** 2020-02-10

**Authors:** Mihailo Miljanic, Anna Capasso, Todd A. Triplett, S. Gail Eckhardt, Kyaw L. Aung

**Affiliations:** 1 Department of Oncology, The LIVESTRONG Cancer Institutes and Dell Medical School, The University of Texas at Austin, Austin, TX, USA

**Keywords:** Clinical trials, combination strategies, gastrointestinal cancers, immune checkpoint inhibitors, primary resistance

## Abstract

Immunotherapy is a rapidly evolving treatment paradigm that holds promise to provide long-lasting survival benefits for patients with cancer. This promise, however, remains unfulfilled for the majority of patients with gastrointestinal (GI) cancers, as significant limitations in efficacy exist with immune checkpoint inhibitors (ICIs) in this disease group. A plethora of novel combination treatment strategies are currently being investigated in various clinical trials to make them more efficacious as our understanding of molecular mechanisms mediating resistance to immunotherapy advances. In this article, we summarize the current status of immune checkpoint blockade in GI cancers and discuss the biological rationales that underlie the emerging treatment strategies being tested in ongoing clinical trials in combination with ICIs. We also highlight the promising early results from these strategies and provide future perspectives on enhancing response to immunotherapy for patients with GI cancers.

## Introduction

Gastrointestinal (GI) cancers collectively represent the most common cancer worldwide and the second leading cause of global cancer deaths.[1] In the USA alone, 160,820 people died from GI cancers in 2018, and for patients under 50 years of age, the incidence of GI cancers has increased by 22% between 1995 and 2013.[1,2] Notably, colorectal cancer (CRC) is the second leading cause of cancer deaths in the USA at present, while the incidence and mortality of hepatocellular carcinoma (HCC) and pancreatic cancer (PC) have also risen. By 2030, PC, CRC, and HCC, respectively, will become the second, third, and fourth leading causes of cancer deaths in the USA.[3] Thus, GI cancers are a major public health burden, and new therapeutic strategies are urgently needed to improve survival outcomes.

Beyond conventional therapies, immune checkpoint blockade has now emerged as a new treatment paradigm across multiple solid tumors that could potentially provide long-lasting survival benefits.[4,5] However, in GI cancers, the major benefit of immune checkpoint inhibitors (ICIs) is mainly limited to those with microsatellite instable (MSI) high tumors, whereas patients harboring microsatellite stable (MSS) tumors remain virtually unresponsive. Even though subsets of patients with HCC and esophageal and gastric cancers with a high programmed death ligand-l (PD-L1) combined positive score (CPS) derive clinical benefit from ICIs, the benefits remain relatively modest compared to those seen in other cancers that are more responsive to ICIs such as melanoma, lung cancer, and renal cell carcinoma.

To make immunotherapy more efficacious in GI cancers, sound biological understanding of the primary and secondary resistance mechanisms to immunotherapy in this disease group will be necessary. Critical too will be the development of predictive companion diagnostic biomarkers to select patients for immunotherapy. In this article, we review the current status of ICIs in GI cancers and discuss the key biological mechanisms impeding immunotherapy efficacy in GI cancers together with the emerging novel strategies being employed in ongoing clinical studies in combination with ICIs to overcome resistance.

### Current Status of Immune Checkpoint Blockade in Gastrointestinal Cancers

The most promising results with ICIs in GI cancers are observed in patients with MSI high tumors. A single-arm, Phase II trial evaluated pembrolizumab, a programmed death 1 (PD1) inhibitor, as a second- or third-line agent in 41 advanced cancer patients (11 with MSI high CRC; 21 with MSS CRC; and 9 with MSI high non-CRC).[6] The objective response rate (ORR) was 40% (95% confidence interval [CI], 12–74) in the MSI high CRC group and 0% (95% CI, 0–20) in the MSS CRC group. The progression-free survival (PFS) was significantly higher in MSI high group at 20 weeks (78% [95% CI, 40–97] vs. 11% [95% CI, 1–35]). The ORR was 71% (95% CI, 29–96) in MSI high non-CRC patients.[6] These early results were confirmed in a followed-on study that investigated pembrolizumab in 86 patients with advanced MSI high cancers who failed first-line therapy, including 66 patients with MSI high GI cancers (40 with CRC; 8 with PC; 5 with small intestinal cancer; 5 with gastroesophageal cancer; 4 with cholangiocarcinoma; and 4 with ampulla of Vater carcinoma).[7] The ORR was 52% (95% CI, 36%–68%) in MSI high CRC patients and 54% (95% CI, 39%–69%) in MSI high non-CRC patients.[7] Based on these results, pembrolizumab was granted approval by the Food and Drug Administration (FDA) to be used as a second-line agent in advanced MSI high cancers including GI cancers. However, it is noteworthy that patients with MSI high tumors represent only a small subset of patients with GI cancers, especially in PC, where a recent study found that only 0.8% of patients (7/833 patients screened) had MSI high tumors, highlighting the lack of immunotherapy option for almost all patients with PC.[8] In this study, four of the seven MSI high PC patients (57%) achieved treatment benefit (1 complete response, 2 partial response [PR], and 1 stable disease [SD]) from pembrolizumab, underscoring the clinical efficacy of PD1 blockade in MSI high PC.[8]

Nivolumab, a PD1 inhibitor, was also studied either alone or in combination with low-dose ipilimumab, a CTLA-4 inhibitor, in patients with advanced MSI high CRC in a second- or third-line setting. In the Phase II, multicohort, checkmate-142 trial, 74 patients received nivolumab monotherapy, whereas 1194 patients had nivolumab plus ipilimumab.[9] In the monotherapy cohort, at a median follow-up time of 12 months, 31% (95% CI, 20.8–42.9) of the patients achieved an objective response.[9] While median duration of response was not reached at the time of report, eight (11%) patients had responses lasting 12 months or longer (Kaplan–Meier 12-month estimate: 86%, 95% CI, 62–95).[9] In contrast, with the combination, at a median follow-up time of 25.4 months, 6% (*n* = 7) of the patients achieved complete response and 62% (*n* = 52) achieved PR.[10] The median duration of response was again not reached with 68% of responses ongoing at the time of data cutoff.[10] Two-year PFS and overall survival (OS) rates were 60% and 74%, respectively.[10] However, 31% of the patients experienced Grade 3–4 treatment-related adverse advents (TRAEs) and in 13%, treatment was discontinued due to TRAEs.[10] These results underscore the efficacy of single-agent PD1 blockade in MSI high CRC, and demonstrate the significant additional benefit achieved with the combined PD1 and CTLA-4 blockade at the expense of toxicities. Based on the results of the Checkmate-142 trial, nivolumab and nivolumab plus low-dose ipilimumab were approved by the FDA for use in second-line setting for patients with MSI high advanced CRC who progressed following treatment with fluoropyrimidine, oxaliplatin, and irinotecan.

Currently, there is no approved ICI for MSS CRC, PC, small-bowel carcinomas, and cholangiocarcinoma. However, the results from ICI studies are emerging in MSS CRC and PC. Based on the promising results from a Phase Ib trial,[11] the Phase III IMblaze370 trial investigated the efficacy of a combination of the PD-L1 inhibitor atezolizumab and a MEK ½ inhibitor, cobimetinib, in comparison with atezolizumab alone or regorafenib monotherapy in patients with chemorefractory advanced MSI stable CRC.[12] No differences in ORR, PFS, or OS between the treatment groups were observed.[12] The randomized Phase II CCTG CO.26 trial, on the other hand, demonstrated a superior survival with the combination of durvalumab, a PD-L1 inhibitor, and tremelimumab, a CTLA-4 inhibitor, in patients with advanced chemorefractory CRC when compared to best supportive care (OS 6.6 vs. 4.1 months, hazard ratio [HR]: 0.72 [90% CI, 0.54–0.97], *p* = 0.07).[13] These results were the first to show that combined immune checkpoint blockade prolonged survival in advanced CRC not selected for MSI status. In PC, a Phase 1b trial investigated the efficacy of pegylated interleukin 10 combined with FOLFOX chemotherapy in the second-line setting and reported a median OS of 10.2 months that is superior to historical controls.[14] An ORR of 10% and a 6-month disease control rate of 13% were demonstrated in another Phase Ib trial that evaluated the combination of the anti-colony-stimulating factor (CSF) CSF1 receptor antibody cabiralizumab with nivolumab in 31 advanced PC patients who had prior chemotherapy.[15] Three confirmed PRs were in patients with MSS tumors. The combination of gemcitabine and nab-paclitaxel chemotherapy with a novel CD40 agonistic monoclonal antibody APX005M with or without nivolumab was also investigated in treatment-naïve metastatic PC patients in a recent Phase Ib study.[16] Of 24 evaluable patients, 83% (*n* = 20) experienced tumor reduction, with PRs observed in 56% (*n* = 14). These early results do indicate that novel immunotherapy combination strategies could produce promising antitumor activity in patients with PC.

Of 23 advanced esophageal cancer (EC) patients treated in the KEYNOTE-028 trial, which investigated the efficacy of pembrolizumab in advanced solid tumor patients with a PD-L1 CPS score of ≥ 1 (CPS is determined by the number of PD-L1 staining cells in a tumor sample divided by the total number of viable cells, with the result multiplied by 100), who failed to respond to first-line therapy, ORR of 30% (95% CI, 13–53) was observed with a median duration of response of 15 months (range, 6–26).[17] The KEYNOTE-059 trial, on the other hand, assessed ORR and clinical response (CR) to pembrolizumab in 259 patients with gastric and esophago-gastric junction cancers in the third-line setting.[18] The ORR was 15.5% (95% CI, 10.1–22.4) in patients with PD-L1-positive tumors, while they were 6.4% (95% CI, 2.6–12.8) in patients with PD-L1-negative tumors.[18] The median duration of response was also higher in the PD-L1-positive group (16.3 months [range, 1.6+–17.3+] vs. 6.9 months [range, 2.4–7.0+]).[18] Based on the results of the KEYNOTE-059 trial, pembrolizumab was approved to be used in the third-line setting in patients with advanced gastric and gastroesophageal junction cancers with a tumor PD-L1 CPS score of ≥ 1.

The Phase III KEYNOTE-062 trial recently evaluated pembrolizumab plus chemotherapy or pembrolizumab alone versus chemotherapy in the first-line setting in HER2-negative gastric and gastroesophageal junction cancers with a PD-L1 CPS score of ≥ 1.[19] No improvement in OS or PFS was observed in pembrolizumab plus chemotherapy group. However, pembrolizumab monotherapy was reported to be noninferior to chemotherapy, based on the primary endpoint OS results, and was better tolerated even though PFS was shorter in the pembrolizumab group (2.0 vs. 6.4 months [HR: 1.66, 95% CI, 0.37–2.01]). Furthermore, crossover of OS curves was observed at 12 months, indicating that a subgroup of patients (at least 30%) treated with pembrolizumab monotherapy had a shorter OS compared to chemotherapy group.[19] Despite this, pembrolizumab alone offers noninferior OS compared to chemotherapy with an improved safety profile and as such should be considered for first-line treatment in this patient population. Subgroup analysis demonstrated a clinically meaningful OS benefit in patients with a tumor PD-L1 CPS score of ≥ 10 treated with pembrolizumab (median OS 17.4 vs. 10.8 months, HR: 0.69 [95% CI, 0.49–0.97]). Similarly, in the single-arm, Phase II KEYNOTE-180 trial, ORR of 20% (95% CI, 8.0–37.0) was observed with pembrolizumab in 35 patients with squamous cell EC with a PD-L1 CPS score of ≥ 10.[20] Furthermore, the KEYNOTE-181 randomized Phase II trial evaluated pembrolizumab as a second-line therapy in patients with advanced EC and showed an improved survival with pembrolizumab in those with squamous cell histology and a PD-L1 CPS score of ≥ 10 compared to standard chemotherapy (OS 10.3 vs. 6.7 months, HR: 0.64 [95% CI, 0.46–0.90], *p* = 0.0074).[21] Of note, pembrolizumab did not improve OS in all randomized patients when compared to chemotherapy.[21] Based on the results of KEYNOTE-180 and KEYNOTE-181 studies, pembrolizumab was recently approved for use in patients with advanced squamous cell ECs with a PD-L1 CPS score of ≥ 10 in the second-line setting.

PD1 inhibitors, nivolumab and pembrolizumab, are also approved for use as a second-line standard treatment for patients with HCC. The Phase I/II Checkmate-040 study evaluated the efficacy of nivolumab in both sorafenib-naïve/intolerant patients and patients who had disease progression on sorafenib therapy with advanced HCC and Child-Pugh Class A status.[22] An ORR of 20% (95% CI, 15–26) was observed in 214 patients treated in the dose-expansion cohort.[22] Notably, these responses occurred regardless of the etiology or tumor PD-L1 expression.[22] The median PFS was 5.4 months (95% CI, 3.9–8.5), with 6 months and 9 months OS of 89% (95% CI, 77–95) and 82% (95% CI, 68–90), respectively.[22] The Checkmate-040 trial also included a separate cohort of patients with Child-Pugh Class B (B7–B8) status (*n* = 49); 25 sorafenib naïve and 24 sorafenib experienced.[23] The ORR was 10.2% in this population with a median duration of response of 9.9 months and the median OS was 7.6 months.[23] Four patients (8.2%) had hepatic select TRAEs and only two (4.1%) discontinued treatment due to TRAEs underscoring the safety of nivolumab in this population.[23] On the other hand, the results from the KEYNOTE-224 trial showed an ORR of 17% (95% CI, 11–26) with pembrolizumab in sorafenib-experienced patients (80% of participants had disease progression on sorafenib and 20% discontinued sorafenib due to intolerance) with advanced HCC (94% of patients had Child-Pugh Class A status).[24] The median time to response was 2.1 months (interquartile range: 2.1–4.1). The median duration of response was not reached (range, 3.1–14.6 + months), but 77% of the responders showed a response for at least 9 months.[24] A higher PD-L1 CPS score was associated with a higher ORR and longer PFS.[24] However, despite these promising results, the Phase III KEYNOTE-240 trial that compared pembrolizumab with best supportive care as second-line therapy in 433 patients with advanced HCC failed to show statistically significant improvement in OS and PFS compared to best supportive care.[25] Similarly, the CheckMate-459 Phase III study that evaluated nivolumab as first-line treatment in patients with advanced HCC in comparison with the standard sorafenib therapy failed to meet the prespecified primary OS endpoint.[26] This clearly highlights the need for better patient selection strategies using high precision biomarkers. The accelerated approvals of pembrolizumab and nivolumab by the FDA in the second-line setting were conditional on Phase III confirmation, and it is yet to be determined whether the current FDA label of these agents will remain in light of results from these studies.

Encouragingly, recent data are emerging to support combining PD1/PD-L1 blockade with anti-angiogenesis agents for better efficacy in patients with advanced HCC. In a recent Phase 1b trial of multikinase inhibitor lenvatinib plus pembrolizumab in patients with unresectable HCC, four patients achieved complete response (6.0%), 26 PR (38.8%), and 25 SD (37.3%).[27] However, serious adverse events were observed in 42 (62.7%) patients. These initial results are indeed promising for efficacy but concerning for toxicities. Most recently, the results from the Phase 3 IMBrave150 trial that compared atezolizumab plus bevacizumab to sorafenib in the first-line setting in patients with advanced HCC were reported.[28] Significantly improved median OS (not estimable vs. 13.2 months [HR: 0.58, 95% CI, 0.42–0.79, *p* = 0.0006]), median PFS (6.8 months vs. 4.5 months, 95% [HR: 0.59, 95% CI, 0.47–0.76, *p* < 0.0001), and ORR (27% vs. 12%, *p* < 0.0001) were observed in the atezolizumab plus bevacizumab arm.[28] Based on these results, this combination may now represent the new standard of care in the first-line setting for advanced HCC. The current FDA-approved ICIs in GI cancers and their indications are summarized in Table 1.

**Table 1:** Food and Drug Administration-approved immune checkpoint inhibitors in gastrointestinal cancers and their current indications

**Agent (dose)**	**Cancer type**	**PD-L1 status**	**FDA-approved indication**
Pembrolizumab (200 mg every 3 weeks)	Any	Any	Patients with unresectable or metastatic MSI-high or mismatch repair-deficient tumors that have progressed following prior treatment and who have no satisfactory alternative options or colorectal cancer that has progressed following treatment with fluoropyrimidine, oxaliplatin, and irinotecan[7]
	Esophageal	CPS ≥ 10	Patients with recurrent locally advanced or metastatic squamous cell carcinoma of the esophagus whose tumor express PD-L1 (CPS ≥ 10) as determined by an FDA-approved test, with disease
	Gastric	CPS ≥ 1	progression after one or more prior lines of systemic therapy[20,21] Patients with recurrent locally advanced or metastatic gastric or gastroesophageal junction adenocarcinoma whose tumors express PD-L1 (CPS ≥ 1) as determined by an FDA-approved test, with disease progression on or after 2 or more prior lines of therapy including fluoropyrimidine- and platinum-containing chemotherapy and if appropriate, HER2-targeted therapy[18]
	HCC	Any	Patients with HCC who have been treated with sorafenib[24]
Nivolumab (240 mg every 2 weeks or 480 mg every 4 weeks)	Colorectal	Any	Patients with MSI-high or mismatch repair-deficient metastatic colorectal cancer that has progressed following treatment with a fluoropyrimidine, oxaliplatin, and irinotecan[9]
	HCC	Any	Patients with HCC who have been previously treated with sorafenib[22]
Nivolumab plus ipilumumab (nivolumab 3 mg/kg followed by ipilimumab 1 mg/kg on the same day every 3 weeks for 4 doses, then nivolumab 240 mg every 2 weeks or 480 mg every 4 weeks)	Colorectal	Any	Patients with MSI-high or mismatch repair-deficient metastatic colorectal cancer that has progressed following treatment with fluoropyrimidine, oxaliplatin, and irinotecan[10]

CPS: Combined Positive Score, HCC: Hepatocellular carcinoma, MSI: Microsatellite instability, FDA: Food and Drug Administration, HER 2: Human epidermal growth factor receptor

## Emerging Strategies to Improve Immune Checkpoint Inhibitors' Efficacy in Gastrointestinal Cancers

**Figure 1:**
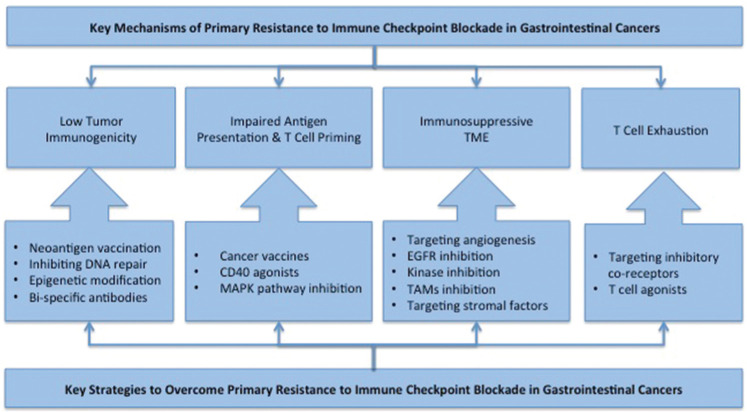
Summary of key primary resistance mechanisms to immune checkpoint blockade and key strategies that are being employed to overcome the resistance in gastrointestinal cancers. EGFR: Epidermal growth factor receptor, MAPK: Mitogen activated protein kinase pathway, TAM: Tumor associated macrophages, TME: Tumor microenvironment.

Mounting an immune response to tumors by host is a multistep process and theoretically, any deficiency in this process could lead to tumor immune escape.[29] Based on advances in our understanding of biological mechanisms that underlie the immune evasion in GI cancers, new treatment strategies are being developed and tested in ongoing clinical trials. Herein, we discuss the key primary resistance mechanisms to immune checkpoint blockade in GI cancers, together with the emerging strategies that are being employed to improve the clinical outcomes achieved with ICIs in this disease group [Figure 1]. Table 2 summarizes the current Phase 2 and Phase 3 clinical trials that are testing the efficacy of combining ICIs with key therapeutic strategies to overcome the immunotherapy resistance in GI cancers.

**Table 2:** Ongoing Phase 2 and 3 combination clinical trials with immune checkpoint inhibitors in advanced gastrointestinal cancers

**Trial number**	**Phase**	**ICI**	**ICI pathway**	**Combination therapy**	**Combination strategy**	**Cancer type**	**Patient selection**	**Status**
**Enhancing tumor immunogenicity, antigen presentation, and T**-**cell priming**								

NCT03190265	2	Nivolumab, ipilimumab	PD1, CTLA-4	GVAX/CRS-207, cyclophosphamide	Cancer vaccine, chemotherapy	Pancreatic cancer	None	Active, recruiting
NCT03836352	2	Pembrolizumab	PD1	DPX-survivac, cyclophosphamide	Cancer vaccine, chemotherapy	Hepatocellular carcinoma, MSI high GI cancers	Survivin expression (not applicable to colorectal and gastric cancers)	Active, recruiting
NCT01896869	2	Ipilimumab	CTLA-4	Allogenic GM-CSF transfected pancreatic tumor vaccine	Cancer vaccine	Pancreatic cancer	Patients must achieve stable disease after 8–12 doses of folfirinox	Active, not recruiting
NCT03806309	2	Nivolumab	PD1	OSE2101	Cancer vaccine	Pancreatic cancer	Patients must achieve stable disease or partial response after 8–12 doses of folfirinox	Active, recruiting
NCT03006302	2	Pembrolizumab	PD1	GVAX/CRS-207, epacadostat, cyclophosphamide	Cancer vaccine, IDO inhibitor, chemotherapy	Pancreatic cancer	None	Active, recruiting
NCT03161379	2	Nivolumab	PD1	GVAX, SBRT, cyclophosphamide	Cancer vaccine, radiotherapy, chemotherapy	Pancreatic cancer	Patients with borderline resectable disease only	Active, recruiting
NCT02648282	2	Pembrolizumab	PD1	GVAX, SBRT, cyclophosphamide	Cancer vaccine, radiotherapy, chemotherapy	Pancreatic cancer	Patients with locally advanced disease only	Active, recruiting
NCT03723915	2	Pembrolizumab	PD1	Reolysin	Oncolytic virus	Pancreatic cancer	None	Active, recruiting

**Targeting oncogenic signaling**								

NCT03201458	2	Atezolizumab	PD1	Cobimetinib	MEK inhibition	Biliary tract cancer	None	Active, not recruiting
NCT02060188	2	Nivolumab, ipilimumab	PD1, CTLA-4	Cobimetinib	MEK inhibition	Colorectal cancer	None	Active, not recruiting
NCT03608046	2	Avelumab	PD1	Cetuximab, irinotecan	Anti-EGFR antibody, chemotherapy	Colorectal Cancer	BRAF wild type, MSS	Active, recruiting
NCT03409848	2	Nivolumab, Ipilimumab	PD1, CTLA-4	Trastuzumab	Anti HER-2 antibody	Gastric cancer and gastroesophageal junction cancer	HER2 positive disease	Active, recruiting
NCT03818997	2	Atezolizumab	PD1	DKN-01, paclitaxel	Wnt pathway inhibition, chemotherapy	Esophageal cancer, gastric cancer, gastroesophageal junction cancer, and biliary tract cancer	None	Not yet recruiting

**Targeting tumor microenvironment**								

NCT03895970	2	Pembrolizumab	PD1	Lenvatinib	Anti-angiogenesis	Hepatocellular carcinoma and biliary tract cancer	None	Active, recruiting
NCT02519348	2	Durvalumab, tremelimumab	PD-L1, CTLA-4	Bevacizumab	Anti-angiogenesis	Hepatocellular carcinoma	None	Active, recruiting
NCT03841201	2	Nivolumab	PD1	Lenvatinib	Anti-angiogenesis	Hepatocellular carcinoma	None	Active, recruiting
NCT03713593	3	Pembrolizumab	PD1	Lenvatinib	Anti-angiogenesis	Hepatocellular carcinoma	None	Active, recruiting
NCT03439891	2	Nivolumab	PD1	Sorafenib	Anti-angiogenesis	Hepatocellular Carcinoma	None	Active, Recruiting
NCT03603756	2	SHR-1210	PD1	Apatinib, irinotecan/paclitaxel/nedaplatin	Anti-angiogenesis, chemotherapy	Esophageal cancer	SCC	Active, recruiting
NCT03951597	2	JS001	PD1	Lenvatinib, gemcitabine, and oxaliplatin	Anti-angiogenesis, chemotherapy	Biliary tract cancer	None	Active, recruiting
NCT03414983	2, 3	Nivolumab	PD1	Bevacizumab, FOLFOX (fluorouracil, leucovorin, and oxaliplatin)	Anti-angiogenesis, chemotherapy	Colorectal Cancer	None	Active, not recruiting
NCT02997228	3	Atezolizumab	PD1	Bevacizumab, FOLFOX (fluorouracil, leucovorin, and oxaliplatin)	Anti-angiogenesis, chemotherapy	Colorectal Cancer	MSI High	Active, Recruiting
NCT03698461	2	Atezolizumab	PD1	Bevacizumab, FOLFOX (fluorouracil, leucovorin, and oxaliplatin)	Anti-angiogenesis, chemotherapy	Colorectal cancer	Patients with potentially resectable liver metastases only	Active, recruiting
NCT02873195	2	Atezolizumab	PD1	Bevacizumab and capecitabine	Anti-angiogenesis, chemotherapy	Colorectal cancer	None	Active, not recruiting
NCT03336216	2	Nivolumab	PD1	Cabiralizumab	CSF1R inhibition	Pancreatic Cancer	None	Active, recruiting
NCT03694977	2	PDR001	PD1	MCS110	CSF1R inhibition	Gastric cancer	None	Active, recruiting
NCT03331562	2	Pembrolizumab	PD1	Paricalcitol	Vitamin D analog	Pancreatic cancer	Patients who achieved stable disease or partial response for a period of 2 months with no further shrinkage of ≥30% on scan on their first-line chemotherapy	Active, recruiting

**Overcoming T-cell exhaustion**								

NCT03642067	2	Nivolumab	PD1	Relatlimab	LAG-3 inhibition	Colorectal Cancer	MSS	Active, Recruiting
NCT02060188	2	Nivolumab, ipilimumab	PD1, CTLA-4	Daratumumab, anti-LAG-3 antibody	CD38 inhibition, LAG3 inhibition	Colorectal cancer	None	Active, not recruiting
NCT03662659	2	Nivolumab	PD1	Relatlimab, XELOX (oxaliplatin, capecitabine)/FOLFOX (fluorouracil, leucovorin, oxaliplatin)/XOS (oxaliplatin, S-1)	LAG3 inhibition, chemotherapy	Gastric cancer and gastroesophageal junction cancer	None	Active, recruiting
NCT03680508	2	TSR-042	PD1	TSR-022	Anti-TIM3 antibody	Hepatocellular carcinoma	None	Not yet recruiting

**Epigenetic reprogramming**								

NCT03250273	2	Nivolumab	PD1	Entinostat	Histone deacetylase inhibitor	Biliary tract cancer, pancreatic cancer	None	Active, recruiting

Trials that combine ICIs with chemotherapy alone were not included in this table. ICI: Immune checkpoint inhibitors, MSI: Microsatellite instability, MSS: Microsatellite stable, GI: Gastrointestinal, CTLA: Cytotoxic T-lymphocyte antigen, IDO: Indoleamine 2, 3-dioxygenase

### Enhancing tumor immunogenicity

One of the major roadblocks to the efficacy of ICIs in MSS GI cancers is that they are poorly immunogenic with a low tumor mutation burden (TMB) (< 50/genome).[30] It is known that a high TMB is associated with increased neoantigens that can be presented by major histocompatibility complexes (MHC) on tumor cells,[31] resulting in immune recognition and cytotoxic T cell responses. This is best exemplified by deep immune responses seen with ICIs in MSI high tumors that contain an average of over 1000 somatic mutations per genome, which is nearly twenty times that of MSS tumors.[30,32,33] However, it is still unclear whether a high TMB is a prerequisite for responses to immunotherapy. There is a subset of patients with MSS CRC tumors with low TMB that have high immunoscore, defined by densities of intratumoral CD3 + and CD8 + cytotoxic T cell infiltrates and an independent prognostic factor of patient outcomes independent of MSI status, indicating that these patients may still benefit from immunotherapy.[34]

One particular approach to address low tumor antigenicity is utilizing a personalized neoantigen vaccination approach to augment the existing antitumor T cell responses, either alone or in combination with other strategies. Inhibiting DNA repair may also lead to an increase in neoantigen generation and expression by tumor cells and enhance recognition by the immune system.[35,36] Supporting this, previous preclinical studies in CRC have demonstrated that the combination of PARP inhibitors with ICIs resulted in synergistic increase in CD8 + T cell infiltration and antitumor activity.[37] Furthermore, DNA fragments resulting from double-stranded breaks may enter the cytoplasm and activate STING-dependent Type I interferon responses, augmenting immune checkpoint blockade.[37] Epigenetic drugs are also of particular interest to increase the expression of cancer-associated antigens including tumor testis antigens and viral antigens.[38,39] Both hypomethylating and histone modification agents have been shown to induce this effect in preclinical studies.[39,40]

Strategies to bypass traditional T cell activation through recognition of MHC: Ag complexes are also currently being evaluated to improve CR. This includes bi-specific antibodies that simultaneously bind to surface tumor-associated antigens and CD3 on T cells, leading to their activation and proliferation. Clinical trials are currently evaluating carcinoembryonic antigen (CEA)–T cell bi-specific (TCB) antibody directed toward CEA, a protein that is often overexpressed on the surface of CRC tumor cells.[41,42] Notably, CEA–TCB is the first TCB antibody that showed preliminary efficacy in the treatment of solid tumors, including patients with MSS stable CRC.[42] Clinical trials are currently ongoing, and adoptive cell therapy of engineered cells expressing chimeric antigen receptors toward CEA is also being investigated in patients with metastatic CRC.[43] Furthermore, adoptive T-cell transfer therapy targeting mutant KRAS G12D was shown to produce effective antitumor activity in a patient with metastatic CRC, indicating early promise for this approach.[44] Interestingly, adoptive transfer of mutation-reactive TH1 CD4 + T cells recognizing mutated erbb2 interacting protein ERBB2IP in a patient with metastatic cholangiocarcinoma achieved an initial decrease in target lesions followed by a prolonged period of SD.[45] Upon progression, re-treatment resulted again in the regression of metastatic disease, suggesting that this approach holds promise in epithelial tumors.[45]

### Improving tumor antigen presentation

The second molecular roadblock to ICIs in GI cancers is the deficient cancer antigen presentation through MHC downregulation.[46] Constitutively, active oncogenes such as mutant KRAS downregulate MHC expression effectively, impeding tumor-associated antigen presentation.[47] Thus, strategies to increase the presentation of existing neoantigens through modulating MHC expression or enhancing the ability of antigen-presenting cells (APCs) to activate T cells could be of greatly benefit. Therapeutic cancer vaccines are of particular interest to enhance tumor antigen presentation by APCs, especially dendritic cells (DCs), to boost the effector cytotoxic T cells' response to tumor and induce long-lasting tumor-specific T-cell-mediated immunity and memory. There are multiple ongoing studies combining ICIs with therapeutic cancer vaccines in GI cancers [Table 2]. Approaches with a genetically engineered herpes virus vaccine are of particular interest due to their ability to mediate tumor lysis, enhance loading of peptides onto MHC Class I molecules for presentation by tumor cells, as well as express granulocyte-macrophage CSF in order to enhance DCs' activity.[48] Listeria-based vaccines have also shown promise in several preclinical models for stimulation of both innate and adaptive immunity and DC priming.[49]

Targeting oncogenic pathways using kinase inhibitors may also increase MHC expression and antigen presentation. To this end, there is strong preclinical data to support that targeting MAPK pathway using MEK inhibitors increased tumor neo-antigen expression, intratumoral effector T cell infiltration, and synergy with PD1 blockade in CRC models.[50,51] Nonetheless, combining cobimetinib, a MEK inhibitor, with atezolizumab, a PD-L1 inhibitor, did not produce meaningful clinical benefit in patients with chemorefractory CRC in a recent Phase III study.[12] However, combining MEK inhibitors with other immunomodulatory agents remains a potential therapeutic strategy to bolster immune response in this setting.

### Targeting tumor microenvironment

The tumor microenvironment (TME), consisting both of adaptive and innate effectors, plays a vital role in determining response to immunotherapy. For instance, MSI high CRC tumors had increased enrichment of cytotoxic CD8 + T-cells, natural killer (NK) cells, Th1 helper T-cells, and a greater degree of Th17 T-cell activation in comparison to MSS counterparts.[52] Moreover, MSI high tumors have an underenrichment of T-regulatory cells.[52] In contrast, immunosuppressive TME predominates in MSS GI cancers. Enrichment of myeloid-derived suppressor cells (MDSCs) is seen in TME across MSS GI tumors and associated with poor prognosis.[53–55] MDSCs play an immunosuppressive role through the suppression of T-lymphocytes and NK cells as well as by upregulating regulatory T-cell activity.[56] Supporting this, elevated levels of circulating MDSCs or enrichment of MDSCs in tumors has been shown to be associated with a poor survival across GI cancers.[53,57,58] Furthermore, tumor-associated macrophages (TAMs) play immunosuppressive role in GI cancers by inhibiting NK cell activation as well as by promoting tumor angiogenesis and stimulating MDSCs.[59,60] TAM infiltration was associated with a poor response to treatment and worse prognosis in EC, PC, and HCC.[61,62] It was also shown that cancer-associated fibroblasts (CAFs) promote polarization of TAMs toward immunosuppressive protumoral phenotype,[63] contributing to immunosuppressive TME. Furthermore, growing evidence indicates a tumor-promoting role of CAFs and TAMs in the bile duct epithelium and cholangiocarcinoma.[64,65]

Modulation of the immune TME in order to reduce immunosuppressive mediators and enhance cytotoxic activity has long been proposed as a viable strategy to combine with ICIs. Currently, there are several trials investigating this approach in GI cancers. Inhibiting CD73 and CD39 is of particular interest. CD73 has a dual role in T-regulatory self-reinforcing autocrine stimulation as well as paracrine suppression of effector T-cell activity within the TME.[66] Considering the immunosuppressive role of TAMs in GI cancers,[67] targeting TAMs using CSF1R inhibitors is of intense interest in the field and this approach is producing early clinical promise in PC.[15]

Targeting tumor angiogenesis may also reverse immunosuppressive TME to enhance ICIs. Beyond the direct antitumor effect of anti-angiogenesis agents, studies have suggested a synergistic relationship between anti-angiogenesis agents and ICIs.[68,69] Anti-angiogenesis treatment leads to normalization of tumor vasculature and alleviation of hypoxia in the TME.[70,71] Importantly, vessel normalization was shown to decrease the recruitment of immunosuppressive cells such as MDSCs and regulatory T-cells,[72] whereas increasing polarization of macrophages to M1 phenotype resulted in augmented antitumor response.[73] Preclinical *in vivo* studies using colon adenocarcinoma mouse models have demonstrated a synergistic antitumor effect of anti-angiogenesis and PD1 blockade,[74] lending a biological basis for combination of these agents with ICI in CRC. Encouragingly, a recent Phase Ib study demonstrated a promising early efficacy of PD-L1 inhibitor atezolizumab combined with anti-angiogenic agent bevacizumab in systemic treatment-naïve, advanced HCC with an ORR of 62%.[75] Indeed, the superiority of this combination over sorafenib in the first line setting has now been demonstrated in a confirmatory phase 3 study.[28]

Interestingly, EGFR inhibitors were shown to induce favorable immune activation through increase in intratumoral cytotoxic T-cell activity and suppression of T-regulatory and MDSC function in preclinical models.[76] Other kinase inhibitors including mesenchymal–epithelial transition (MET) inhibitors, PI3K inhibitors, and regorafenib are also being tested to improve responses to ICIs in CRC as well as HCC through modulation of immune TME. MET, a marker of drug resistance in many tumor types, promotes EMT transition and angiogenesis and inhibits DC maturation.[77] However, its potential immune-stimulatory role as a tumor-associated antigen makes the biological basis for combining MET inhibition with ICI complex.[77,78] Meanwhile, PI3K inhibition was shown to enhance CD8 + T-cell antitumor response and reduce T-regulatory, TAM, and MDSC activities within the TME.[79,80] Preclinical *in vivo* studies have shown that knockdown of PI3K in colon cancer models can lead to reduced MDSCs and decreased tumor growth.[80,81] Furthermore, regorafenib, a multitargeted kinase inhibitor, was also shown to increase tumor CD8 + T cell infiltration and enhance immunotherapy response in murine models.[82] Supporting this, a recent study has demonstrated an encouraging antitumor activity of regorafenib plus nivolumab in patients with advanced MSS CRC (*n* = 7; ORR 29%) and gastric cancer (*n* = 11, ORR 44%) in a Phase Ib study.[83] Lastly, strategies to target factors made by the tumor and surrounding stroma hold promise in augmenting antitumor T cell responses. This includes targeting the indoleamine 2, 3-dioxygenase with small-molecule inhibitors and engineered enzymes that degrade kynurenine, an immunosuppressive metabolite produced during tryptophan depletion shown to dampen responses to immunotherapy in CRC models.[84,85] Targeting transforming growth factor (TGF) -β is of also particular interest given its role in modulating the balance of T-regs and Th17 cells in the gut.[86] In direct support of this, combining OX40 agonists with a TGFβ inhibitor caused regression of large established tumors in preclinical CRC models.[87]

### Overcoming T cell exhaustion

Tumor-reactive T cells are often exhausted as a consequence of chronic TCR stimulation in the absence of co-stimulation that is characterized by unresponsiveness. During this time, different co-receptors become upregulated that correlates with the degree of exhaustion, which may differ between distinct TMEs. Thus, identifying appropriate co-receptors expressed by tumor infiltrating lymphocytes may be necessary for overcoming T cell exhaustion in addition to PD1 to enhance cytotoxic antitumor immune responses. To this end, LAG3, TIM-3, and CD38 are emerging targets [Table 2]. Previous studies have shown that LAG-3, a known T cell exhaustion marker, may act synergistically with PD1 to inhibit immune activation, and dual LAG-3 and PD1 blockade has demonstrated exciting clinical efficacy in metastatic melanoma patients who are resistant to PD1/PD-L1 blockade.[88,89] TIM-3 is an immune checkpoint that could be co-targeted with PD1 or CTLA4 blockade to overcome T cell exhaustion.[90] However, TIM-3 has context-dependent stimulatory as well as inhibitory function on T cells,[91] and it was also shown to be dispensable for T cell exhaustion.[92] As such, results from ongoing preclinical and clinical studies will be critical to determine the expression of targetable co-receptors on T cells in divergent tumor types in order to refine strategies to achieve optimal therapeutic responses. Emerging data have also indicated that tumors develop resistance to PD1/PD-L1 blockade by upregulating CD38 that inhibits effector T cell function through adenosine receptor signaling and inhibiting CD38 overcomes resistance in tumor models.[93] Based on promising results in the preclinical studies, CD38 antibodies have now entered early-phase clinical trials.

### Epigenetic reprogramming

Epigenetic modifiers hold potential to reprogram tumor as well as immunosuppressive cells in TME for therapeutic advantage. For example, through modification of histone and nonhistone proteins, histone deacetylase inhibitor (HDACi) has a wide range of effects that include induction of apoptosis, cell cycle inhibition, vascular function, and immunomodulation.[94] The role of HDACi in immunomodulation has been controversial, with some studies indicating possible expansion of T-regulatory populations following treatment,[95] whereas others suggesting promising enhancement of immune-stimulatory mediators and antitumor response.[94,96,97] Notably, addition of etinostat to PD1 blockade in preclinical *in vivo* metastatic PC models has demonstrated increase in activated cytotoxic T-cell activity, suppression of MDSCs, and significantly improved tumor-free survival. Although the mechanisms by which HDAC inhibition can result in immunomodulation have yet to be fully elucidated, recent preclinical data suggest that combination with ICI may be a promising avenue for enhancement of antitumor immune response.[98,99]

### Future Perspectives

Considering the current status of immune checkpoint blockade in GI cancers, progress must be made in order to augment ICI efficacy. Beyond MSI status, and PD-L1 positivity, few other predictors of CR currently exist. As such, most current clinical trials do not employ patient selection strategies beyond these markers. A significant proportion of MSI high patients still demonstrate a lack of response to ICIs alone and in combination with other regimens. Greater understanding of the biological basis behind responders and nonresponders within MSI high patient populations may aid in determining which tumor markers and components of the TME play a pivotal role in rendering tumors sensitive or insensitive to ICIs. While this would be beneficial for expanding the subset of MSI high patients who benefit from ICIs, it may also expose molecular mediators in the MSI stable microenvironment that underlie the lack of response to ICIS.

Elucidating differences in specific neoantigen epitope expression and their qualities is an exciting proposition. However, attempts to do this thus far have demonstrated that there are thousands of different neoantigens that may be expressed, and that only exceedingly few of these are consistently observed between different tumors even within the same cancer type.[100] This being the case, research efforts that instead focus on the elucidation of the currently unknown or poorly understood mechanisms of immune suppression by tumor cells beyond PD1 and CTLA-4 pathways may be of significant value. Such approaches have already yielded exciting new understanding regarding mechanisms of immune escape and have revealed novel targets to combine with ICIs such as Dickkopf-related protein 2 in the context of CRC.[101]

The current literature consistently revealed key immune-suppressing mediators in TME of GI cancers. Among these are immature DCs, MDSCs, TAMs, regulatory T-cells, and CAFs. Two distinct strategies could potentially be used to address these roadblocks. First, recruitment of new immune-activating mediators to the tumor site may aid in shifting the microenvironment balance in favor of responsiveness. Multiple trials are already testing this hypothesis clinically. Second, reprogramming already-present immune suppressive cells within the TME into immune-activating mediators may shift the balance of stimulating versus constraining components in favor of a more robust immune response.

Among the most exciting combination strategies are those utilizing ICIs with the addition of immunomodulators such as LAG3 inhibitors, TIM3 inhibitors, and CD73 antibodies. A preponderance of preclinical data suggests that these approaches may act synergistically with immune checkpoint blockade. Therapeutic cancer antigen vaccines may still have a role in combination with other agents due to their potential for more efficient immune priming. Lastly, epigenetic modifiers have the potential to reprogram immunosuppressive cells in the TME into therapeutic advantage. However, this remains speculative at this juncture, and increased understanding of the role of different epigenetic modulators, their targets, and precise effects of these drugs on the immune TME will be necessary before their true potential is fully realized.

## Conclusion

The current status of immunotherapy in GI cancers reveals that efficacy has been largely limited to patients with MSI high tumors and a small subset of patients with MSS tumors. Thus far, no ICIs have been approved for patients with MSS pancreatic and CRCs, the two most common causes of GI cancer-related deaths in the USA. Although there are emerging treatment strategies to make immunotherapy more effective in GI cancers, the apparent lack of patient selection strategies beyond using PD-L1 and MSI status hampers progress in this field. Further molecular insight into the heterogeneity of tumor intrinsic as well as extrinsic mechanisms mediating resistance to ICIs will be key in making significant advances in enhancing response to ICIs in GI cancers.
